# Flagellation of the Child’s Back Previous to Its Complete Delivery, as a Preventive of Uterine Hemorrhage, Etc.

**Published:** 1880-02

**Authors:** Isaac E. Taylor

**Affiliations:** Emeritus Professor of Obstetrics and Diseases of Women, and President of Bellevue Hospital Medical College of New York, etc.


### The Practitioner.

VOL. I.——*February, 1880.*——NO. 2.

ARTICLE I.

### 1. FLAGELLATION OF THE CHILD'S BACK PREVIOUS TO ITS COMPLETE DELIVERY, AS A *PREVENTIVE* OF UTERINE HEMORRHAGE.

### 2. FLAGELLATION OF THE ABDOMEN OF THE WOMAN AFTER THE DELIVERY OF THE PLACENTA, AS A *SUBSTITUTE* FOR THE INTRODUCTION OF THE HAND INTO THE UTERINE CAVITY OF THE UTERUS.

READ BEFORE THE NEW YORK ACADEMY OF MEDICINE, FEBRUARY 5, 1880,

BY ISAAC E. TAYLOR, M. D.,

Emeritus Professor of Obstetrics and Diseases of Women, and President of Bellevue Hospital Medical College of New York, etc.

MR. PRESIDENT AND FELLOWS OF THE ACADEMY OF MEDICINE: I most cheerfully assent to the wish and action of the Obstetrical Section requesting me by resolution to present the views and opinions which I laid before them December 23, 1879, for the consideration of the Fellows of the Academy this evening.

The title of my paper is embodied in two propositions:

*First*—Flagellation or spanking the child's back previous to its complete delivery, as a *preventive* of uterine hemorrhage.

*Second*—Flagellation of the abdomen of the woman after the delivery of the placenta, as a *substitute* for the introduction of the hand into the uterine cavity.

We will all admit the physiological fact, that the uterus is the only organ in the female economy that has an habitual sanguineous fluid issuing from it. We also know that it is the only organ which physiologically has large oblique open sinuses without valves, the blood from these sinuses coming directly from the vena cava and the heart itself, and not coming from the returning veins of the uterus.

The slightest derangement, either from a physiological or a pathological process, in the separation of the maternal from the fœtal circulation, may entail an unfavorable and sometimes a fatal termination. Frequently not the slightest evidence is given before or after labor has commenced. Everything in the lying-in chamber, before and after delivery of the child, appears to be progressing favorably; the countenance of the mother is radiant with joy, and that of the attendants and the medical man cheerful and encouraging, when the blood is suddenly heard gushing forth in a full and rapid stream, and the patient is in a state of extreme syncope.

Again: we are congratulating ourselves on the auspicious event, the safe delivery, the expulsion of the placenta, and the first admonition we have of danger is the trickling of blood from the bedside of the patient; our patient faintly calling for fresh air and help; the "besoin de respirer" manifested, gasping and sighing; the pallor of a puerperal hemorrhage; the uterus flaccid, almost imperceptible to the touch, and wrinkled like a bit of old rag, as Caseaux says, when the hand has been introduced into its cavity. A wonderful change has suddenly and instantaneously come over their glad and happy dreams. The bright, cheerful and elated home is changed into the sombre, despondent and melancholy one of apparent dissolution, and it may be of death itself.

The graphic and emphatic declaration of Burns in these sudden and alarming cases, is true: that we are virtually contending with death. Appreciating the perilous condition of our patient, there is no safety or security whatever for her, except from Nature's tourniquet, instantaneous uterine contraction.

Blundell has seen two cases die suddenly in one night from this cause. In cases of this decided character, though not frequent, it is imperative that the obstetrician should be provided with all possible resources, and they should be employed for the welfare of his patient. He should possess in himself calmness, courage, judgment, decision, promptness of action; and if not thus fortified mentally and prepared, he should never, as Lee has said, "cross the threshold of the lying-in chamber."

At the meeting of the American Gynecological Society, held in Philadelphia, September, 1878, two papers on the "Treatment of

Post-partum Hemorrhage" were read and presented for consideration. A long discussion ensued respecting the different methods of treatment in those cases. One of the papers—that by Dr. Wilson, of Baltimore—advocated the hand as a curette to remove all or whatever portions of the placenta that may remain, and to excite uterine contraction by scraping the inner surface of the uterus. The other paper was by Prof. Penrose, of Philadelphia, who recommended very highly, after several years experience, the introduction of a rag or pocket handkerchief, saturated with common vinegar, into the uterine cavity and squeeze it. Both of these papers had reference to, and were suggestive of, treatment by art after the delivery of the placenta.

From the nature of the remarks which were made on that occasion, I am induced to present and suggest another method or means to the many already before the profession and so generally pursued. I am fully aware that it might seem almost superfluous for me to even attempt or hint another method, but the favorable results arising from it prompt me to do so. It is one, however, simple, efficient and decided. One always on hand and at hand, having for its recommendation a physiological basis, not only as a means for arresting the blood or flooding in many cases decidedly after the delivery of the child; but secondly, it is especially of more and greater importance as an aid to prevent the flooding from taking place before and after the delivery of the placenta. I shall consider the method of treatment which I present, as I said, in two propositions:

*First*—Flagellation or spanking the child's back moderately, every now and then, after the delivery of the shoulders, permitting the breech and the extremities of the child to remain in the vagina, and the feet thus placed in apposition with or in the cervix uteri, remaining for fifteen or twenty minutes or more without being withdrawn. Pressure over the uterus by the hand is to be avoided till the delivery of the child, which should be slow and gradual, as it might effect the delivery of the child before we have gained our object, and at the same time the spanking should be quick but gentle, and not too harsh, and continued until the delivery of the child is completed.

*Second*—After the delivery of the placenta, should hemorrhage occur, expose the abdomen, and flagellate it with a towel doubled up, the ends held in the hand, saturated or not with ice water. Several rapid and powerful strokes should be made, when the unrecognized uterus will be almost immediately felt contracting or contracted, no matter how profuse or rapid the flow may be. In one instance, having ocular demonstration after the delivery of the placenta, the stream of blood was as large, full and rapid as that which flows from a Croton faucet.

Should uterine contraction ensue and relaxation take place, a milder application of the same means may be resorted to, till the contraction is deemed secure, and other measures adopted, if necessary.

There can be no procrastination or temporizing action in these sudden and violent cases. The appearance of the method to those present, or to the patient herself if conscious, with the suddenness and rapidity of its application, may seem harsh, abrupt and unnecessary. We have, however, nothing to do with appearances or feelings in such critical emergencies. We are imperatively reminded that life or death is swaying in the balance. Duty commands decided and prompt action. By this procedure I have in some instances had the gratification of feeling the apparently lifeless organ fold itself up under the touch, the uterus contracting or contracted, and our patient's life safe certainly for the time being. Under such circumstances, hot or cold water injections, as well as the hand internally, has in many instances failed to arouse into contraction the perfectly atonic or moribund organ.

After contraction has once been secured, then that treatment which the views or experience of the medical attendant may elect can be pursued, whether by hot water or cold, externally or internally, or mixed with other substances, or by tincture iodine or sulphate of iron, accompanied with the ordinary and usual manipulations externally over the uterus.

In multipara we have the history of the case to guide us with reference to the impending danger, and we should be on the lookout for the great emergency in order to prevent its occurrence. In primipara we may fear the loss of blood consequent on the idiosyncrasy of the patient to flooding; a prolonged and feeble labor; an over-distension of the uterus from the size of the child or excessive liquor amni; a too rapid delivery; a want of regular and perfect contraction, or a malarious condition of the general system, and the round, globular body of the uterus is not felt after the delivery of the child.

I have given some attention to the literature respecting the procedure I am advocating in both propositions and principles, and have failed to find any authority for the execution of it in the same manner, to the same extent, and for the same physiological object. I am not unmindful of all the physical agents usually adopted, and the manipulations of the abdomen over the uterus, but I do not propose to refer to them, as they are admitted to be effective in many of the ordinary cases with which we are all conversant.

In the few remarks I shall offer, I shall confine myself solely and entirely to the primary hemorrhage. I bar out those cases associated with fibrous tumors, polypi, or adhesions to the peritoneum, which are rare, and which have to be treated according to the peculiarities of the case. I also exclude those cases which come from secondary causes, such as anemia, heart or kidney disease, which are to be treated also according to the nature of the case.

I refer especially in this paper to uterine atony or inertia produced from any cause, and presenting a functional paresis or sopor of the uterus, appearing sometimes before, but more especially after the delivery of the placenta.

The method of treatment after the delivery of the child or after the delivery of the placenta is addressed to—

*First*—Those cases where the uterus is large, soft and flabby, and scarcely recognized by the hand.

*Second*—Those where the uterus is distended with blood, and as large as at full term of gestation.

*Third*—The smaller number, the soft uterus, and less perilous.

I mean that form when the uterus acquires the disposition of becoming rapidly firm, then spontaneously relaxing itself and bleeding freely, alternating in this way more or less for some time.

It is an old and trite adage that "an ounce of prevention is worth a pound of cure." In the treatment of uterine hemorrhage we are not to forget the physiologico-pathological condition of the uterus, the great changes the muscular structure has undergone during gestation to prepare it for its important mission, and also the nature of the labor. Its muscular structure may be thicker in some portions than others, or the whole uterus may be insufficiently developed, as I have witnessed in some post-mortem cases, resembling the dilatation with attenuation seen in some cardiac cases, or we may have a perfect atrophy of that part of the uterus where the placenta has been attached, and when bared there will be a jutting in consequent on a more or less paresis of that part of the uterus, or it may be quite the reverse, and fully one-half inch thick and firm.

We must also take into consideration the peculiar anatomical arrangement of the uterine sinuses, flat and thin, and without valves, freely anastomising with the oblique openings at the floor of the vessels, and closely and intimately imbedded in the muscular structure. The blood therefore issues directly from the vena cava and the heart itself, the life of the patient resting solely on the uterine contraction which follows after the delivery of the child. If this contraction is not promptly performed, which as a general rule it is, then the integrity of this structure consequent on an excess of physiological ramollissment greater or less, wholly or partially is lost, the sinuses will not be closed, atony occurs, or a functional paresis, accompanied or associated as it is with a general feebleness or exhaustion of the nervous system. It will not be difficult, therefore, to comprehend why the blood should flow forth in such an appalling and rapid manner as it does sometimes. But relaxation of the uterus may exist without hemorrhage, though we may not be able to give any satisfactory explanation of the cause, for I have seen two cases in one day, but certainly the sinuses must have been closed sufficiently to have prevented blood from flowing at the time. Conversely, hemorrhage may be present when the uterus is firmly contracted. For these kinds of cases therapeutical agents and other physical means are appropriate, as the hemorrhage may come from some lesion of the vagina, vulva or cervix, or a thrombosis. Should the cause of the relaxation with flooding be from portions of the placenta which remain after the hand has been introduced, and failed to arrest the hemorrhage, then the fingers used as a curette, as suggested by Dr. Wilson, will materially aid, and naturally so, in conducting the case to a favorable termination with reference to the flooding by removing the remnants. Valuable as this method is, and absolutely necessary in some important cases, the curetting or scraping or tearing off the remnant, especially after delivery of the placenta, will, I think, require great care, caution, gentleness of touch and tact in the execution of it.

As bearing on this point at this time, I recall while a student in Philadelphia with my brother a post-mortem specimen he obtained, having been in consultation with a medical friend. An attempt was made to remove the placenta for the purpose of arresting hemorrhage, and although carefully and gently executed by the attending physician, such was the softness and tenacity of the uterine tissues, that a perforation took place and the patient died a short time afterward.

Several authors admit the unfortunate event. This case made a decided impression on my mind at that time, and I have always carefully avoided, as we are not certain what the exact status of the muscular structure is, introducing the hand into the uterine cavity (although I know many do it frequently and sometimes in almost every case), as for partial or complete adhesion of the placenta, and which, from my own experience, I should say was rare. Entertaining the physiological views I do as to the management of the placenta, and with a little patience and the conduct of the labor at the termination in the delivery of the child, I have had no occasion to insert my hand into the uterus for the delivery of the placenta except three times in thirty-five years, and then it was for complete adhesion of that organ to the parietes of the uterus.

The usual manipulations of the uterus after the delivery of the child, and sometimes before, we are all well acquainted with, as laid down by obstetrical authorities, and they are usually and generally carried out faithfully. Nevertheless I shall avail myself of the present opportunity to refer to two or three of them only, as I do not desire to burden my paper with many references, for it is intended to be a short and practical one, simply.

Joseph Clarke, of Dublin, whose views and method are so frequently referred to, and time sustained, I will by way of remembrance intrude on your memory once more:

"When," he says, "the head and back of the child are expelled, follow down the fundus of the uterus in its contraction by your hand on the abdomen until the fœtus is entirely delivered, and continue this pressure for some time afterward.

"This was the practice insisted on by that eminently practical obstetrician, Gooch, and he considered it as applicable to cases where the uterus showed a tendency to imperfect action in expelling the fœtus, and where the same imperfection may extend to the expulsion of the placenta."

Dr. Osborn, with a view to securing perfect contraction and preventing retention of the placenta and hemorrhage, advised the retardation of the delivery of the child after the birth of the head, by resisting the rapid expulsion of the shoulders and body, even in cases the most natural. I intentionally cite these few older authorities as their views and experience have been handed down to us, and still actuate the minds of many of the profession with slight modifications. As a course of practice, they illustrate the great care that was taken by them in this particular part of obstetrical practice.

Objections have been made to Clarke and Osborn's methods, but more especially the latter. Ingleby considered "That to retard the descent of the child in ordinary obstetricy was highly objectionable, since this would be substituting a process of art for a process of Nature."

Other authorities consider delay in the delivery of the child as being unnatural, and tending to impair the powers of the uterus, inducing irregular contraction, if not endangering the integrity of the organ to such an extent as to produce or lead to hemorrhage.

As touching on retardation, or delay of the child in its delivery, I will incidentally refer to a case related by Ingleby himself, who was so much opposed to delay, and which is in some measure corroborative of one of the propositions or principles I suggest, and negatives his express views on that practice. Dr. Ingleby says: "The three former labors of Mrs. S—— had terminated very speedily, and at the birth of the child the flooding in each case was so excessive as to lead to the conviction that death would have ensued had the attending physician not been at the bedside. The labor on this occasion, from malposition of the head, proved more tedious, and an instrumental one, and the gradual and slow delivery of the child, separated the placenta without any hemorrhage whatever."

Of all the physical agents the introduction of the hand into the uterine cavity, and carrying it up to the fundus, not only to remove the placenta, but particularly to act as a direct stimulus and excitor of the uterine nerves going to the body of the uterus, is admitted to be one of the oldest, most ready, important and decided means for arresting the flooding, and inducing contraction of that organ, for safety and security to the woman. For this special object, some prefer the closed fist placed in direct contact with the spot where the placenta was attached; others, internal and external pressure at the same time, and thus compressing the organ between their hands; others, flapping their fingers against the parietes of the uterus; others, introducing into the uterus sponges or cloths saturated with different fluid astringent substances, and some the packing of the uterus with sponges and cotton, or handkerchiefs, or an empty ox bladder, and then filling it with water. Miller, of Louisville, Kentucky, reiterates, again and again, the imperative necessity of using the hand in these cases. "The hand, the hand," he says, "is the main and chief chance for the woman, by being introduced into the cavity of the uterus for her safety and security." Gooch recommends that when once the hand has been introduced it should not be removed till contraction has occurred. This, I believe, is the course usually adopted and executed by many of the profession both here and abroad. The practice, nevertheless, is not and has not been accepted or adopted by all obstetricians. Our highly distinguished and celebrated Dewees was decidedly opposed to this method. He says, "He has not found it necessary to introduce the hand, for the purpose of stopping uterine hemorrhage after the placenta is expelled, for thirty-five years, and he regarded it as frightfully unnecessary and pernicious." He further remarks: "Because you have heard the plan recommended, you will, without a moment's reflection, pass your hand into the cavity of the uterus after the expulsion of the placenta, on the first occasion that you have had the opportunity of doing so, and remove all the coagula, then rub the inner surface of the uterus with your fist till you are tired, but without effect. Carry your hand, however, no further than the os uteri, which you may with more or less risk, and with greater effect press and rub with the fingers and irritate it, but not the inner surface of the fundus and body of the uterus."

Respecting the latter clause of Dewees' writing, which I have quoted, I shall adduce in corroboration similar testimony from Caseaux: "In case of inertia of the uterus after the removal of the placenta, the most certain and easiest way is direct irritation made over the body and on the neck of the organ by placing the hand on the lower part of the abdomen so as to rub, press and squeeze the uterine walls, while at the same time the two fingers are passed into the vagina to irritate or titillate the os uteri."

These are the only two authors I am aware of who have resorted to the manipulation on or to the cervix uteri by special irritation or titillation, but this method was after the delivery of the placenta. They were both chary of the introduction of the hand into the uterus, except for adhesion of the placenta. We might have reason to suppose that Dewees was constrained by fear of thrusting his hand through the cervix uteri or of lacerating it, as that idea seemed to actuate the mind of the profession more at that day or time than at the present.

Dewees and Caseaux went no further with the hand than the cervix uteri, and I ask attention to this one essential feature, as it incidentally tends to give support to my own views in one respect with reference to the management of such cases, though previous to the delivery of the child or placenta, and not from the same standpoint or for the same reason. With this method we find Dewees obtained as much, if not more, success than if the hand had been inserted into the cavity of the uterus.

To strengthen these remarks, I must not overlook those of Dr. R. Lee, as one of many others. He asserts: "That he has repeatedly passed the hand into the uterus to produce contraction, but it has refused to obey the stimulus of the hand. It has remained like a soft, flabby bag, more like a piece of intestine than the uterus, and the blood has continued to flow down the arm until it has been withdrawn and more efficient remedies resorted to."

Leroux was also well aware that the stimulus of the hand could not in all cases excite the uterus to contract. He says, "When the os uteri is moderately dilated, and offers no resistance to the introduction of the hand, it will produce no sensation, and the woman will promptly perish from hemorrhage if other means more active and certain are not employed to prevent it."

An important distinction was made by these authorities in cases of uterine inertia, as to the success attending the introduction of the hand to arrest or suppress the flooding. If the os uteri was contracting or disposed to contract, the means, as Levret and Leroux have suggested and adopted, "are very efficacious, and will remove the flooding as if by a charm." But in complete inertia, as stated above, the patient will be likely to die.

The opinion of Burns, whose authority is great on all obstetrical subjects, might be cited as opposed to this distinction, as he affirms that "in every instance this contraction takes place, and that he scarcely introduced his hand into the uterus in a case of flooding without meeting with it, whether the placenta had or had not been expelled." Burns in this opinion stands alone, and I need add no corroborative proof independent of remarking that the general opinion is at variance with it.

Lee was especially a strong advocate for pressure externally, and he recommended it as the most powerful of all other means. With reference to these cases he remarks, "If the hemorrhage is constant, powerful and forcible pressure on the fundus of the uterus, before and after labor, with the two hands should be made. This is an outlook to the Crede method. Hardy and McClintock, of Dublin, on this method of delivering the placenta and arresting the flooding, say: "If the amount of contraction be not sufficient to repress the hemorrhage—that is, before the placenta is cast off—it will be necessary to expel the placenta from the cavity of the uterus. We have been surprised at the ease with which the placenta was pressed out during a contraction of the uterus, when previously it has withstood our best efforts." The clear enunciation and decided exposition of this method by expressions as far back as 1845, in the management not only for the delivery of the placenta but to prevent hemorrhage, antedates and has prior claims, and justly so, to Crede, so much referred to by some members of the profession at this day as his.

The different views and opinions and experiments regarding the physical method of the introduction of the hand into the cavity of the uterus, for preventing and arresting uterine hemorrhage at the time of parturition, leads me to a brief exposition of the method I have adopted for a number of years, the physiological reasons why, and the results founded on them.

We must not be unmindful that the uterus is a vital machine, and all the ingenuity and contrivance of Nature's handiwork in its mechanism to close the uterine sinuses to prevent hemorrhage before or after labor is and must be subordinate to, and directed by, the vital principle which regulates its action at this important crisis in a woman's life. It is not solely as a vital machine we have to consider it; it is as a living organ. The regulation of the function of the nervous system, general or reflex, we have to remember, and especially the latter, in the powerful influence that system has in sustaining or repressing the efficiency of that mechanism of structure to which I have alluded. The positive and direct relationship between the muscular contraction and the nervous power is so intimate and important that a defect in one will necessarily be a defect in the other.

Should the physiological changes in the muscular structure be in excess at the close of gestation, accompanied with an impairment of the general nervous system, the muscular contraction will be insufficient to shut up the venous sinuses, and flooding will follow. Should the nervous power be too feeble or weak to impart due force to the muscular structure, the uterus will be left in a state of stupor or functional paresis, and an unfavorable issue will be the sequence.

The contractility of tissue is in some instances feeble, but it may be and is of sufficient force or power to be of great advantage for the safety of the patient, but in other instances it is altogether wanting, and death ensues. The physiological action and functions of the body of the uterus and its cervix were not comprehended as clearly in a neurological aspect by the authors I have quoted as at the present day. Even now they are not regarded as much, as the investigation of the nervous system indicates. Extensive investigations of the nerves going to the uterine organs and their connections have been made, their claims recognized and their correctness sustained. The practical exemplification of the cases I shall present ratifies, I think, these physiological investigations and experiments through the physical method instituted, and the favorable results which have followed, for preventing and arresting the sudden and copious floodings from the uterus in the class of cases mentioned.

It is an established and conceded fact that in the dilating stage of the cervix uteri during labor, the nerves engaged are those which are solely and wholely in relation with the true spinal marrow or medulla spinalis. The uterus is contracting and dilating alone. It is simply the medulla spinalis which is in action. The medulla oblongata, or the functions over which that portion of the nervous system presides, is not as yet engaged. We all know that when the fœtal cranium comes to pass through the cervix uteri and the head of the child to press upon the vaginal surfaces, a new series of events arises and another class of excitor nerves begin to be set in motion: the reflex spinal action is instituted, and to this is added the respiratory element, a powerful and valuable force.

*Again:* After the delivery of the child, it is a recognized fact, that the tender surfaces of the vagina and cervix uteri become more instantly excitor, and when they are touched the motor irritation of the uterus will be more susceptible and quickly provoked and more rapid than when acting alone. The vaginal and the cervical excitor nerves are not only in relation to the true spinal marrow, or medulla spinalis, but they are in consonance with the medulla oblongata and cerebellum. The excitation of the vaginal and cervical nerves, therefore, through reflex action, arouses the motor nerves going to the body of the uterus, and they become instantaneously engaged, or brought into action, by the irritation or titillation of the child's feet when the back is slapped, aided through the distension of the vagina by the breech of the child.

The physiological views presented and so generally accepted respecting the innervation of the uterus, have lately been investigated and attested by Hauck of Halle, by numerous experiments on animals, affirming that the nerve centres regulating the uterine contractions are centered in the cerebellum, medulla oblongata, and the lumbar portion of the spinal cord. We recognize, therefore, that superficial excitation electrically of the cerebral convolutions causes no uterine contraction.

Excitation of the vagus, when alone, has no effect on the uterus.

Excitation of the cord, when the sympathetic and the sacral nerves are divided, has no effect on the uterus.

Excitation of the uterus itself calls forth only local muscular movement, and no strong general contraction of the organ. Unless, therefore, the reflex nervous system can be brought into action, when the general nervous system has suffered or become prostrated through a tedious and prolonged labor, or idiosyncrasy of constitution or exhaustion consequent on profuse flooding, giving rise, as it will in some cases, to almost complete anemia of that organ—if, under these circumstances, we cannot rouse into action that vitally important system, and produce contraction of the uterus quickly and promptly, but rest content with the direct method, or irritation through the uterine nerves almost solely, whether by external or internal means, an unfavorable issue dependent upon the want of excitation, whatever that may be, of the reflex system will occur, and the patient will be most likely to die. I do not forget that another external and important physical agent is resorted to sometimes, originally suggested by Gooch—that is, cold water dashed or poured from a height, on the abdomen when exposed—one to all appearance closely allied in principle to the method I propose. The shock, however, produced by this means may be so transient as to create no permanent effect in some cases. I will not advance my own experience on this point, but give that of one of the most eminent and practical obstetricians on this subject. The flow of blood, he remarks, may be arrested for a time, and relaxation may follow after a short interval, and the hemorrhage be renewed again with equal violence as at first, and we cannot with propriety expect it the second time to be of any value.

I should do injustice to the subject under consideration if I made no allusion to the application of hot water, at a temperature of 110 to 115 degrees F., injected into the uterine cavity as an hemostatic. Various opinions are entertained regarding the success of this method and how to apply it. Some believe that its benefit results as a direct stimulus or excitor, the same as any other liquid application; others, from their experience, believe that it should be classed as a styptic, the same as iodine or the different preparations of iron, and producing thrombosis of the venous sinuses. Dr. Athill, of Dublin, advises its direct application to the fundus. Dr. Albert H. Smith, of Philadelphia, only beyond the internal os uteri. By some it is considered efficient and of great value. By others its failure has been realized. When the placenta had been cast off, its success was very marked. When the placenta or portions of it remained, it proved of no value, nor did the hot water induce uterine contractions, leading to their expulsion, according to Gussereau. Jackell considers its greatest efficacy is in purely atonic hemorrhage, and that form of relaxing and contracting alternately. As a remedy for promptness of action, it has its proper cases for election. Valuable as it is, however, the delay consequent on not having the instrument on hand and at hand in many instances, except by anticipation, may be too great, and the patient succumb in the meantime or die a few hours after. If the rationale of its success is not as a styptic, producing thrombosis of the uterine sinuses, then it must be considered among the remedies acting through the direct method or excitation of the uterine nerves solely, and the reflex system is not brought into play, it will fail, as well as the introduction of the hand.

Electricity is a remedial agent, suggested many years ago by Radford, of Manchester, who gave more *eclat* to this means of arresting hemorrhage consequent on uterine inertia than any practitioner previous or since. The principle is in consonance, in one or two points, with the physiological views expressed, and there is no excitor or stimulus more energetic to produce muscular contraction; none has a more powerful influence over a torpid or moribund uterus. It is a remedy which, as a rule, is not present for instantaneous use, and it has not been applied in the extreme cases. It was and has been more especially resorted to after the hemorrhage has in some measure ceased, or in those cases where relaxation and contraction occur, to establish a more perfect tonic contraction.

Compression of the aorta presents its claims as a means in sudden and profuse floodings, but only after the placenta has been cast off. It has in some measure been prescribed by many, though it has been successful in some few instances. One of the principal objections has been the uncertainty in attempting to make the application of pressure on the vessel itself. The most important method is that of direct pressure above the uterus, while at the same time the hand is introduced into the uterus to press upon the posterior part of its walls, and then on the aorta, and this has to be continued, and has been in some cases, for three or four hours at a time. The same objections apply to this method as to the introduction of the hand, for it is precisely the same, with only the addition of pressure made on the aorta.

As a direct method, I fail to see, from the evidence we have, the prompt benefit resulting from the procedure. Some prominent obstetricians have seen convulsions follow from it, and when adopted it is only as a "dernier resort," and in the last stages of exhaustion the patient never rallies. The reflux action of the blood before contraction has taken place must be in the vena cava and the branches which empty into it. As a secondary agent after contraction has occurred, but the danger not over, as women have died in two or three hours after the flooding has ceased, consequent on the anemia of the cerebrum and spinal marrow, then pressure on the aorta externally will be of great advantage in retarding the blood in the brain, and thus act as a stimulus to that organ to sustain the vitality of the cardiac organ, and the life of the patient prolonged and possibly saved.

With the views I have presented this evening, I concede that a judicious combination of the different methods to which I have incidentally referred, and faithfully carried out, will in many instances induce contraction of the uterus.

But although many agents, therapeutical, physical or mechanical, are employed, only one or two principles, as a general rule, are, I believe, applied, and these, perhaps, from the nature of the case, are not the most important and imperative as the onset of such sudden and critical cases as I have referred to demand. If we do not, then, recognize the high claims which physiology has presented to us through the reflex nervous system, but adopt the direct method alone, and which, I think, is a misnomer, we fail to comprehend the correct principles on which the use of physical and remedial agents act.

CASE NO. 1.—A few years since I was engaged to act in consultation with Dr. G—— in the case of Mrs. V——. Her confinements had been of the ordinary character, accompanied by severe pains, though the labor was not prolonged. This was her fourth confinement. Profuse floodings occurred always after the birth of the child, and the patient was left in a critical state for some time afterward. There was also considerable difficulty in arresting them. The child was above the average weight. I was notified of the event a few hours after the commencement of the labor. When I arrived I was ushered into a room adjoining hers. I was informed shortly after that the labor was progressing favorably—natural presentation and so on. An hour afterward, judging from the strenuous efforts the patient was making and the nature of her pains, the delivery must be near at hand. It was evident if my services were to be of any value, with the views I held, I could be of no assistance in her case unless Nature was more provident this time than on previous occasions. I therefore went into the chamber, and found the head of the infant was born and the shoulders about to be delivered. At this stage I requested, after the shoulders had passed, that the cord should be drawn down, and the breech and extremities of the child detained in the vagina and cervix, the uterus not to be pressed upon nor held. The back of the child was then spanked sharply and quickly for twenty minutes or more, at intervals. There was some slight evidence of hemorrhage, and no more. The child was then slowly delivered. The uterus grasped and well contracted, though not hard. Occasional relaxation and contraction, but no flooding. In ten minutes after the placenta came away safely. Remained for one hour, and everything was natural and safe. Morning visit, all well.

CASE No. 2.—I was requested to attend Mrs. L—— with her fifth confinement, receiving the information how critical her confinements had been, consequent not only on the tedious labor but the excessive flow which followed the delivery of the placenta. The labor was natural, though as formerly tedious for several hours. Chloroform was requested not to be given, and to that I most cheerfully assented. The head of the child was safely delivered by eneucleation, the shoulders came gradually and slowly through the vulva. The cord drawn down; breech and extremities remaining; uterus not held; child's back flagellated occasionally, and not withdrawn for nearly half an hour; every time the child was whipped, the child would gasp. The delay of the delivery of the child created anxiety on the part of the mother, and she asked why the baby was not removed. The sensation of the child's feet she did not like pressing against the womb.

The placenta escaped very soon after the delivery. No flooding ensued but what is incident to delivery in ordinary cases. The getting up was excellent.

Case No. 3.—I was requested many years ago to hasten immediately to meet Dr. Robeson in the case of Mrs. E——, who resided but a short distance from my house, July 4, 1846. She had just been delivered, and the placenta followed soon after. She was blanched, rolling from side to side; gasping, sighing and twitching of the muscles of the face; abdomen as large almost as at full term. The uterus was instantly grasped with the left hand, the right loosing the clots and removing them from the vagina and uterus. Immediately the uterus was flagellated smartly several times, when contraction ensued. This course was pursued moderately and occasionally for several minutes, while the ordinary means were also attended to. No relaxation took place, although she was watched closely for two hours or more. Brandy injections for rectum were given and nourishment. Nothing unfavorable occurred, but a slow convalesence.

Case No. 4.—Mrs. V——. In consultation with Dr. S——, in a case of kyphotic pelvis, having an outlet in the inferior strait of a transverse diameter of $1\frac{7}{8}$ in. between the tuber ischia and antero posterior $2\frac{1}{2}$ inches. After the delivery of the child by cephalotropy, while awaiting the delivery of the placenta, a few moments after the expulsion a rapid and full stream of blood issued from the vagina, as though a water faucet had been opened or turned on. Instantly I arose from my seat and gave the bared abdomen several rapid and strong flagellating strokes over the uterine region with a towel doubled up, wet with ice water, and as instantaneously was the flooding arrested. The round, globular uterus was then felt contracting under the hand. An electric shock could not have acted with more or as much effect so successfully, to the surprise of all present.

In the two cases where syncope seemed almost profound, with a perfectly relaxed uterus, but hemorrhage impending, as that I have already mentioned, and in one of which the husband, being present and at the bedside, exclaimed, "My God, my wife is dead!" the labor was short, quick and severe in a delicate lady, and shock consequent thereon. A dash of two or three tumblerfuls of water, at a distance of several feet, on the face, and a faint sigh was heard, and our hope revived. With gentle flagellation, the relaxed organ was felt to be overcome, and contraction followed. The same principle is here established and sustained in this case by sudden shock as a physical agent at the origin of the reflex system as at its terminus.

Before I close reading my paper, I desire to avail myself of the opportunity, by trespassing for a few moments longer on your good nature, to say a word or two as to the use of anæsthetic agents in cases not only where we know that hemorrhage has occurred, or where we may suspect and dread its taking place, but withholding its application, as a general rule, with only exceptional instances, at the time of the delivery of the child. By this course we may have or obtain a more complete and perfect contraction of the uterus for the casting off of the placenta, and possibly the exemption from flooding. The anæsthetic, from the prolonged sedative influence, in some instances tends materially to impair or produce a functional paresis of the uterus, so much so at times as to arrest its action entirely for several hours, and ergot has to be given to stimulate it into contraction. By this impairment or cessation of uterine action, at the very time when we should require uterine contraction the most, the patient may be subjected more especially to hemorrhage than if we had abstained from its use. When this state of things arises, and though flooding exists, the patient is not capable of responding to any assistance we might require of her, and the time in some cases is long before she comes out from its effects.

Conversely, I can adduce cases where the use of the anæsthetic has been of incalculable advantage when the labor has been severe and sharp, though not long, and the activity of the organ has almost suddenly become exhausted; the labor tending to become tedious; the pains ceasing, but short and jerky and valueless, and everything looking to an instrumental delivery. The anæsthetie in such cases will arouse the tired and fatigued organ from its exhausted or sleepy state, and a few pains will be sufficient to accomplish the birth of the child, no flooding ensuing. Experience, and that considerable, and not the dicta of authority, can only teach us these great practical lessons in the administration of those most invaluable anæsthetic agents. I am fully convinced, and, I may say, that since 1848 I have derived essential benefit and assistance from withholding an anæsthetic at the close or near the termination of the labor. Sleep or drowsiness, consequent sometimes on the exhaustion of the nervous system, sustained by the anæsthetic, is, I believe, dangerous in these cases in various ways, and should be avoided. On the contrary, it is a time when the woman has need of the excitation of wakefulness, united to the assistance of art, to induce the necessary contractility of the uterus, and without which the life of our patient would in some instances flow away with her blood in these sudden and critical cases.

